# A Review of the Efficacy, Safety, and Feasibility of Rifamycin-Based Post-Exposure Chemoprophylaxis for Leprosy

**DOI:** 10.3390/tropicalmed10040084

**Published:** 2025-03-21

**Authors:** Patrick O. Campbell, Nicholas M. Douglas, Stephen T. Chambers

**Affiliations:** 1 Department of Pathology and Biomedical Science, University of Otago, Christchurch 8011, New Zealand; steve.chambers@otago.ac.nz; 2 Department of Infectious Diseases, Christchurch Hospital, Te Whatu Ora Waitaha, Christchurch 8011, New Zealand; nick.douglas@otago.ac.nz; 3 Department of Medicine, University of Otago, Christchurch 8011, New Zealand; 4 Division of Global and Tropical Health, Menzies School of Health Research, Charles Darwin University, Darwin 0810, Australia

**Keywords:** leprosy, chemoprophylaxis, rifamycin, rifampicin, rifapentine, contacts, efficacy, effectiveness

## Abstract

In 2021, the World Health Organization (WHO) recommended scaling up leprosy prevention activities, including chemoprophylaxis, as one of the pillars of their ‘Towards Zero Leprosy’ strategy. This recommendation was primarily based on a 57% overall reduction in leprosy incidence among contacts who received chemoprophylaxis in the COLEP study conducted in Bangladesh. Since this landmark study, further research on the efficacy, feasibility, and implementation of leprosy chemoprophylaxis has been conducted. Additionally, concerns have been raised regarding the strength of evidence supporting the overall benefit of chemoprophylaxis and the potential for propagation of drug resistance in *M. leprae* strains. This literature review presents the current evidence for the efficacy and safety of rifamycin-based chemoprophylaxis in preventing clinical disease, and the feasibility of implementing chemoprophylaxis programmes. Post-exposure prophylaxis (PEP) has a reported efficacy of 45–80%, depending on the degree of case contact, the classification of the index case, the selected chemoprophylaxis regimen, the geographical setting, childhood BCG coverage and the implementation approach. As an intervention, it appears to be feasible, cost-effective, and acceptable to patients, contacts, and healthcare staff, with minimal harm. Implementation strategies need to be tailored to specific epidemiological and sociocultural settings for maximal benefit. Further research is required to optimise PEP regimens and strategies in various epidemiological settings and to assess the impact of these programmes on the susceptibility profile of local *M. leprae* strains.

## 1. Introduction

Leprosy is a communicable, infectious disease caused by *Mycobacterium leprae* (*M. leprae*). It is most likely transmitted via aerosolised secretions from the respiratory tract and has a long incubation period, averaging approximately 5 years (range 6 months to 20 years) prior to the development of clinical symptoms [1]. Leprosy predominantly affects the nerves but can also affect the skin, eyes, joints, and internal organs [2]. The associated loss of sensation and nerve damage can lead to permanent disability and disfigurement, which commonly results in stigmatisation and social exclusion [3]. Following the introduction of multidrug therapy (MDT) in the 1980s, there was a steady decline in leprosy cases reported worldwide, but progress has slowed in recent decades. According to the 2024 WHO global leprosy update, 182,815 new cases of leprosy were reported globally in 2023, representing a 14% decrease in new cases reported during the decade 2014–2023 [4]. The majority (>95%) of new cases were clustered in the 23 global priority countries where the disease remains endemic, with India, Brazil, and Indonesia accounting for almost 80% of total cases [4]. It has become clear that enhanced case-finding and treatment alone will be insufficient to achieve leprosy elimination, and novel approaches are required to interrupt the transmission of *M. leprae*. This is reflected in the most recent WHO leprosy control guidance, where the primary goal has shifted to the elimination of leprosy (defined as the interruption of transmission) through scaling up of leprosy prevention alongside integrated active case detection [5].

Acquisition of leprosy generally requires close, sustained contact with an infectious case. Household contacts (HHC) are at the highest risk, followed by social contacts and those living in close proximity to the index case [6,7]. Given the long incubation period and the likelihood that transmission can occur during subclinical disease, close contacts are an important target population if interruption of transmission is to be achieved. Leprosy elimination is likely to require multiple novel strategies, including improved point-of-care diagnostics for clinical and subclinical disease, development and use of an effective leprosy vaccine, and highly-active chemoprophylaxis regimens for contacts [8]. Post-exposure chemoprophylaxis as a method of interrupting disease transmission was first investigated in the 1960s and 1970s in several randomised controlled trials assessing the efficacy of dapsone and its longer-acting derivative acedapsone, primarily in children of index cases [9,10,11,12,13]. Despite the demonstrated efficacy, this approach was not pursued due to the required duration of therapy, as well as concerns regarding the development of resistance to the primary treatment agent at that time [7,14]. The introduction of MDT for leprosy in the 1980s led to much higher cure rates but despite this, leprosy incidence remained stubbornly high, bringing into question the ability of case-finding and treatment to achieve leprosy control [15]. The availability of newer, more potent antimicrobials temporarily renewed interest in the potential role of leprosy chemoprophylaxis, with the first uncontrolled trial using single-dose rifampicin starting in 1988 [16]. However, an additional 15 years passed with little further research on rifamycin-based chemoprophylaxis. Evaluating the impact of chemoprophylaxis on leprosy transmission was listed as a research priority in the WHO report on leprosy in 2003 [14]. Bakker et al. subsequently reported favourable outcomes with single-dose rifampicin post-exposure prophylaxis (SDR-PEP) using a blanket approach in Indonesia [17]. The safety and feasibility of implementing chemoprophylaxis on a larger scale have since been demonstrated in several studies. [7,18] The COLEP study was the first large-scale study of SDR-PEP in an endemic setting and showed an overall reduction in the incidence of leprosy of 57% in the first two years, setting the platform for further chemoprophylaxis research [7]. At the same time, concerns were raised about the real-world effectiveness of chemoprophylaxis, as well as the possibility of promoting the development of drug resistance and disease-related stigma [19,20].

The aim of this systematic literature review was to evaluate the efficacy, safety, and feasibility of rifamycin-based leprosy chemoprophylaxis for reducing leprosy incidence and to describe the various methods of implementation used to date.

## 2. Materials and Methods

### 2.1. Literature Search

**Figure 1:**
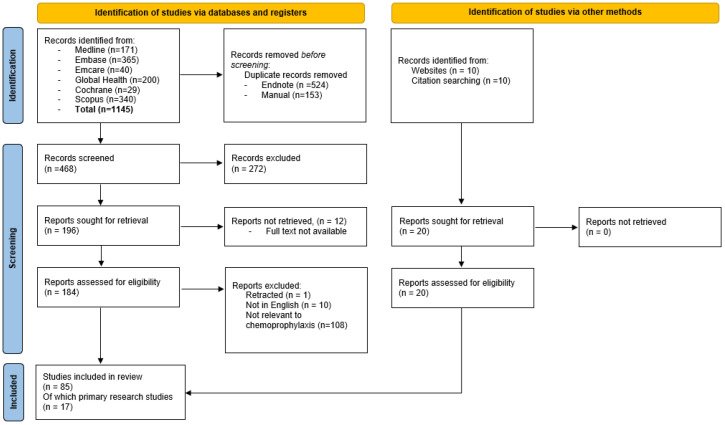
Flowchart of study selection.

Structured searches were conducted on 21 May 2024 from OVID Medline (1946–21 May 2024), Global Health (1910 to 2024 Week 20), Embase (1947–present), OVID Emcare (1995 to 2024 week 20), and the Cochrane and Scopus databases. The search string adapted for each database was “(leprosy OR Mycobacterium leprae OR Hansen’s) AND (Chemoprevention OR chemoprophylaxis OR post-exposure prophylaxis)”. The reference lists of included articles were also searched for additional relevant literature. Duplicates were removed using the referencing software EndNote 21^®^ (Clarivate Analytics, PA, USA) with subsequent manual removal of additional duplicates. A review of retrieved articles was performed, initially of the abstracts, and subsequently the full texts after provisional inclusion. Full-text reviews were conducted by the primary author. Primary research studies on the efficacy of chemoprophylaxis were included if these studies had at least one defined target population that received chemoprophylaxis and clinical or serological responses were subsequently recorded and reported. Studies were not required to have a comparator group or a specified period of follow-up. Randomised controlled trials that are currently recruiting and for which protocols have been published were also included. Exclusion criteria for reports were as follows: language other than English, full-text unable to be obtained, articles that had been retracted, focus on neglected tropical diseases rather than leprosy, not relevant to the aims of this literature review, or focus on other aspects of leprosy prevention and control (Figure 1). No limits were placed on study or article type.

### 2.2. Data Presentation

Due to the marked heterogeneity in study methodology, meta-analysis was not undertaken. Study results are summarised descriptively and in graphical form.

## 3. Results

### 3.1. Literature Review

The literature search produced 1145 potentially relevant articles. After the removal of duplicates and those meeting exclusion criteria, 196 articles remained for full-text review (Figure 1). An additional 20 articles were added following citation searches of the included publications and web searches. Twelve articles did not have full-texts available (five conference abstracts, three review articles, three commentaries, and one report), none of which reported on primary research in the area of interest. Full-text reviews resulted in a further 119 articles being excluded, leaving 85 articles for inclusion (Figure 1). Of the 85 articles included, 17 were primary research articles relating to the efficacy of chemoprophylaxis (eight randomised trials, three implementation studies, three uncontrolled trials, two cohort studies, and one controlled clinical trial), four of which reported on studies that are actively recruiting.

### 3.2. Post-Exposure Chemoprophylaxis

#### 3.2.1. Evidence for Efficacy of Single Agent Rifamycin-Based Chemoprophylaxis Regimens

Studies of the efficacy of rifamycin-based chemoprophylaxis for leprosy are shown in Table 1. The first study reporting on rifampicin chemoprophylaxis was an uncontrolled trial involving population-wide screening and mass administration of single-dose rifampicin (25 mg/kg) in the South Marquesas Islands beginning in 1988. SDR was administered on a single occasion to 2786 (98.7%) inhabitants of the Island and an additional 3144 South Marquesans living elsewhere in French Polynesia. Using previous case detection rates as a historical control, and assuming a 50% reduction in leprosy incidence over the study period unrelated to the intervention, the estimated efficacy of chemoprophylaxis was 40–50% at 4 years [21]. Accounting for an overall reduction in background incidence in the remainder of French Polynesia as a whole, the efficacy at 10 years was revised to 35–40%, which the authors did not feel justified the financial and logistic costs associated with this approach [22]. This study had a number of important limitations, including the lack of a temporally aligned comparator group and small absolute case numbers, leaving the study highly susceptible to time-varying confounders such as independent leprosy control initiatives.

In 2005, a controlled but non-randomised trial of rifampicin chemoprophylaxis, with two doses given approximately 3.5 months apart, was implemented on five Indonesian islands endemic for leprosy [17]. Efficacy was compared across three arms: a ‘control group’ of household and neighbour contacts (n = 1439) who did not receive any prophylaxis, a ‘contact group’ consisting of household and neighbour contacts only (n = 2058), and a ‘blanket group’, consisting of all the inhabitants of 3 islands (n = 1242). Participants in the contact and blanket groups received two doses of rifampicin given approximately 3.5 months apart. A significant reduction in incidence was observed in the blanket group compared with the control group (hazard ratio (HR) 0.28; *p* = 0.031) after 33.5 months of follow-up, but no impact was detected in the contact group, where prophylaxis was only given to household and neighbour contacts. Similar results had been noted in dapsone chemoprophylaxis trials previously [10,23]. These findings suggested a potential role for rifampicin chemoprophylaxis in endemic countries and that blanket approaches may be preferred in these settings.

**Table 1:** Primary research studies on rifamycin-based chemoprophylaxis for leprosy.

First Author	Year Published	Location	Study Design	Study Population	Chemoprophylaxis Regimen	No. of Contacts Included	Duration of Follow-Up	Estimated Efficacy	Comments
Completed rifamycin monotherapy studies									
Cartel [21]	1992	Marquesas Islands	Uncontrolled trial	Endemic population	SDR Rifampicin 25 mg/kg	5895	4 years	40–50%	Small case numbers Susceptible to confounding
Nguyen [22]	2000						10 years	35–40%	
Bakker [17]	2005	Indonesia	Controlled clinical trial	Endemic population (contact group, blanket group + control group)	Two doses of rifampicin 3.5 months apart Adults: 600 mg Children: 300 mg	3965	33.5 months	HR 0.25 (95% CI 0.068–0.95) in blanket group compared to control group (*p* = 0.031)	No difference between contact and control groups (*p* = 0.93)
Moet [7]	2008	India	Cluster randomised controlled trial	Household + neighbour contacts	SDR (weight-based) >35 kg: 600 mg <35 kg: 450 mg Age 5–9: 300 mg	28,092	4 years	57% (95% CI 32.9%–71.9%) overall reduction in incidence at 2 years (*p* = 0.0002)	NNT = 265 (95% CI 176–537) Benefit less in HHC + contacts of MB cases
Khoudri [24]	2018	Morocco	Retrospective time series analysis	Household contacts	SDR (age/weight-based dosing) ≥15 years: 600 mg Age 10–14: 450 mg Age 5–9: 300 mg <20 kg: 10–15 mg/kg	4019 screened 3704 received SDR	12 years	16% per year reduction in NCDR following SDR-PEP introduction (*p* = 0.05)	Multiple limitations Possible confounding
Wang [25]	2023	China	Cluster randomised controlled trial	Household contacts	Single-dose rifapentine or rifampicin >15 years: 600 mg 10–14 years: 450 mg	2331 Rifapentine 2760 Rifampicin 2359 Control	4 years	Cumulative incidence ratio 0.16 (95% CI 0.03–0.87) in rifapentine group compared to control group (*p* = 0.02)	No significant difference in cumulative incidence between rifampicin and control group (*p* = 0.23). No adverse events recorded
Hasker [26]	2024	Madagascar and Comoros	Cluster randomised trial	Arm 1: Control arm Arm 2: HHC only Arm 3: HHC + neighbour contacts (within 100 m) Arm 4: HHC + neighbour contacts + PGL-1 antibody positive	Single double-dose rifampicin (20 mg/kg)	110,666	Madagascar: 2 years Comoros: 3 years	Arm 2 (IRR 0.95, 95% CI 0.4–2.23) Arm 3 (IRR 0.80, 95% CI 0.34–1.87) Arm 4 (IRR 0.58, 95% CI 0.22–1.56) None statistically significant Controlling for baseline prevalence resulted in IRR of 0.56 (95% CI 0.35–0.89) in Arm 3 (*p* = 0.003)	IRR 0.35 (95% CI 0.15–0.82) in HHC on subgroup analysis NNT = 82 for HHC compared to 870 for study population as a whole
Active rifamycin monotherapy studies									
Schoenmakers [27]	Protocol published 2021	Multiple countries	Cluster randomised trial	Endemic districts within countries Health centre-based HHC screening Community-based skin camp intervention	Single-dose rifampicin (150–600 mg)	Calculated sample size: 675 index patients 30,000 contacts	2 years	-	
Coleman [28]	Protocol published 2023	Kiribati	Implementation study	Endemic population Mass drug administration	SDR (Age/weight-based dosing) ≥15 years: 600 mg Age 10–14: 450 mg Age 6–9: 300 mg (>20 kg) Age 6–9: 150 mg (<20 kg) Age < 5 years: 10–15 mg/kg	64,439	3 years	-	
Completed studies of combination rifamycin-based regimens									
Diletto [29]	1999	Federated States of Micronesia	Uncontrolled trial	Endemic population Population-wide, two rounds, 1 year apart	Rifampicin 600 mg, ofloxacin 400 mg + Minocycline 100 mg	105,506 total 90% screened at least once 87% at least one dose ROM	2 years	Impact of chemoprophylaxis on incidence not determined as part of this study	Reduction of cases between subsequent rounds but not possible to separate effects of case finding/treatment from screening/prophylaxis. Relative risk of clinical disease lower in chemoprophylaxis group
Daulako [30]	1999	Kiribati	Implementation study	Endemic population Two rounds of screening nationally 1 year apart PEP administered on: 1st round: Christmas Island only 2nd round: South Tarawa only	Rifampicin 600 mg, ofloxacin 400 mg + minocycline 100 mg	32,645 total for two islands 85% administered chemoprophylaxis	1 year	Impact of chemoprophylaxis on incidence not determined as part of this study.	Good safety profile of regimen with low rates of adverse events.
Tin [31]	1999	Marshall Islands	Implementation study	Household contacts	Rifampicin 600 mg, ofloxacin 400 mg + Minocycline 100 mg	2831 2454 (87%) received chemoprophylaxis	-	Impact of chemoprophylaxis on incidence not determined as part of this study	Good safety profile of regimen with low rates of adverse events.
Khaing [32]	2009	Myanmar	Randomised trial Serological study	Household contacts Control arm Treatment arm	15 years: ROM (dose not specified) <15 years: Rifampicin 15 mg/kg	152 contacts 76 each group	6 months	Efficacy of chemoprophylaxis not assessed as part of this study	Significant reduction in PGL-1 titres in adults but not in children. Non-significant reduction seen overall.
Oo [33]	2008	Myanmar	Randomised controlled trial Serological study	Household and neighbour contacts in endemic community	15 years: Rifampicin 600 mg, ofloxacin 400 mg + minocycline 100mg) <15 years: Rifampicin 15 mg/kg	829 extended contacts	2 years	Efficacy of chemoprophylaxis not assessed as part of this study	Significant reduction in mean antibody titres in adults but not in children 2 years post chemoprophylaxis
Astari [34]	2021	Indonesia	Cohort study	Elementary school children Seropositive PGL-1	Rifampicin 300 mg + Clarithromycin 250 mg once daily for 10/7 + fortnightly for 3 months thereafter	2548 screened 200 seropositive	5 years	No progression to leprosy at 5 years	Significant reduction in PGL-1 antibody titres over study period No comparator group
Active studies of combination rifamycin-based regimen									
Hinders [35]	Protocol published 2023	Multiple countries	PEP ++ Cluster randomised controlled trial	Endemic populations Household contacts Follow-up blanket campaign	Intervention arm: 3 doses Rifampicin 150–600 mg + clarithromycin 150–500 mg four weeks apart Control arm SDR-PEP 150–600 mg	Calculated required sample size of 202,360	2 years	-	
Younoussa [36]	Protocol published 2024	Madagascar + Comoros	BE-PEOPLE cluster randomised controlled trial	Endemic population Household + neighbour contacts (<100 m) Blanket PEP if >50% of village eligible	Intervention arm 2 doses Bedaquiline (400–800 mg) + rifampicin (150–600 mg) four weeks apart Control arm Rifampicin 150–600 mg	Calculated required sample size of 124,000	3 years	-	

Abbreviations: SDR = single dose rifampicin, HHC = household contacts, HR = hazard ratio, NNT = number needed to treat, IRR = incidence rate ratio.

A blanket approach to chemoprophylaxis was also supported by a study in an Indonesian Island in which the entire population of a remote village was screened and offered SDR-PEP if eligible after active leprosy had been excluded. Two rounds of screening and SDR-PEP administration took place at a 1-year interval. 1671 inhabitants (88%) were screened, 1499 (79%) of whom received SDR. The third visit was for the purpose of screening only, during which 1481 people were screened, 410 of whom had not received SDR because of exclusion criteria or absence. The incidence of leprosy was reduced in the group who had received SDR compared to those who had not (OR 0.57, 95% CI 0.16–2.03). This study demonstrated the feasibility and acceptability of this approach in a remote endemic setting [37,38].

Due to the susceptibility of the above studies to various sources of bias, it was acknowledged that evidence from larger randomised trials was required to determine the efficacy of simple chemoprophylaxis regimens and the populations most likely to benefit [14]. The COLEP study was a large cluster-randomised trial conducted in Bangladesh that was designed to determine the efficacy of SDR in close contacts of index cases [7]. Almost 22,000 participants were randomised to receive a single, weight-based dose of rifampicin or placebo after the exclusion of active disease. The risk of incident leprosy in contacts given SDR, as opposed to placebo, was reduced by 57% at 2 years. This effect was sustained with no indication of a rebound effect during the 6-year follow-up period. There was no statistically significant difference in case incidence observed between the rifampicin and placebo groups in the 3rd and 4th years after treatment, possibly due to collateral benefits in the placebo group of regular skin surveys and removal of active cases from circulation through treatment with MDT. An unexpected finding was that unrelated contacts and those living further from the index case benefited more from prophylaxis than close household contacts. It was postulated that this could be due to increased exposure or higher bacillary burden in household contacts (HHC) and therefore reduced likelihood of complete clearance of bacilli with a single dose of rifampicin. While reduced incidence was only statistically significant in extended contacts and contacts of paucibacillary (PB) leprosy in the COLEP study, the protective effect observed in HHC and contacts of multibacillary (MB) cases was approximately 50% (the study was not powered for these outcomes) [7]. This suggested a potentially valuable contribution to clinical disease burden reduction by administering SDR prophylaxis to these groups [39]. A secondary analysis of the COLEP study showed an additive effect of childhood BCG vaccination, increasing the overall protective effect of SDR prophylaxis to 80% in contacts who had received both interventions (95% CI 50–92). This result held true on multivariate analysis when adjusted for age, sex, physical distance to and disease classification of index case [40].

The PEOPLE study was a cluster-randomised trial carried out in Madagascar and Comoros to assess the efficacy of single double-dose rifampicin (20 mg/kg) in reducing leprosy incidence. This study identified a much higher risk of leprosy in household contacts and near neighbours (<75 m) compared with more distant contacts, highlighting the need for active case finding around index cases. No clear benefit over placebo was evident when prophylaxis was limited to household contacts (incidence rate ratio [IRR] 0.95; CI 0.40–2.23), even when adjusted for baseline prevalence (IRR 0.80; CI 0.5–1.29). In the blanket arm, where prophylaxis was extended to neighbour contacts within 100 metres of the index case, the protective effect of SDR achieved statistical significance after controlling for baseline prevalence (IRR 0.56, *p* = 0.003) [26]. The secondary analysis showed a modest protective effect at the individual level (IRR 0.55; 95% CI 0.36–0.83), which was less than that reported in the COLEP trial. However, contrary to the COLEP findings, subgroup analysis showed a higher effectiveness of SDR-PEP in household contacts (IRR 0.35; 95% CI 0.15–0.82). The number needed to treat to prevent one case of leprosy was also significantly lower in this group compared to the study population as a whole (82 vs. 870). The effect size in the blanket arm was less than that observed in the 2005 Indonesian study, which was a population-wide intervention rather than a targeted population who were not geographically isolated. Diagnostic procedures in this trial were shown to be highly reliable, with 87% of leprosy cases confirmed microbiologically by qPCR positivity of skin biopsies.

**Figure 2:**
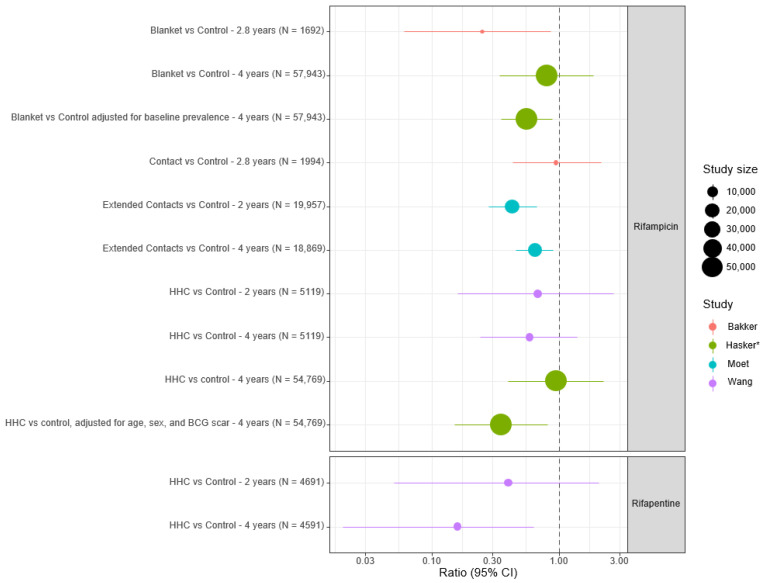
Forest plot summarising findings of controlled rifamycin-based chemoprophylaxis studies. HHC; household contacts; BCG; Bacillus Calmette-Guerin. * Results reported as odds ratios except for Hasker et al., which are reported as incidence risk ratios (IRR).

Rifapentine has several advantages over rifampicin as a potential prophylactic agent. It is more bactericidal in murine leprosy models, has a longer half-life, exhibits lower minimum inhibitory concentrations against *M. leprae* compared to rifampicin, and achieves higher intracellular concentrations [41]. Wang et al. demonstrated superior efficacy in a cluster randomised trial of household contacts in China (cumulative incidence ratio 0.16), with a sustained effect at 4 years, leading to calls for rifapentine to be included in future PEP trials and MDT regimens [25,42]. The longer half-life of rifapentine (T1/2 = 16 hours) may raise additional concerns for the promotion of rifamycin resistance in *M. leprae* and *M. tuberculosis* due to prolonged exposure of organisms to low drug concentrations during elimination. This warrants careful consideration. The findings of the four main controlled clinical studies described above are summarised in Figure 2, and a risk of bias assessment for these studies is included in the supplementary data (Supplementary Figure S1).

#### 3.2.2. Combination/Enhanced Rifamycin-Based Chemoprophylaxis Regimens

Alternative chemoprophylaxis regimens have been trialled in different settings with varying results. In the 1990s, rifampicin combined with ofloxacin and minocycline (ROM) chemoprophylaxis was trialled in the Western Pacific due to persisting high incidence rates of leprosy in several countries. This was generally combined with intensive case finding, MDT treatment, and chemoprophylaxis of contacts. The results of these interventions in the Federated States of Micronesia, Kiribati, and the Marshall Islands were presented at a workshop on leprosy prevention in 1999 [43]. Due to the uncontrolled nature of these studies, it was difficult to distinguish the effect of prophylaxis from that of intensified case finding and MDT treatment. However, this regimen was well tolerated, with no serious adverse effects [29,30]. It was acknowledged that additional research was required to determine the efficacy of this approach, but it was not pursued due to concerns regarding expense and antimicrobial resistance [44]. Administration of single-dose ROM chemoprophylaxis was associated with decreasing PGL-1 antibody titres in close contacts of leprosy patients in two studies from Myanmar. Khaing et al. demonstrated a significant reduction in mean antibody titres in seropositive adults receiving single-dose ROM chemoprophylaxis compared to a non-treatment group (*p* < 0.004). Oo et al. confirmed similar findings in a larger cohort of 849 extended contacts randomised to receive ROM. Participants less than 15 years of age in these studies were treated with single-dose rifampicin rather than ROM, and no statistically significant reduction in antibody titres was observed in this subgroup. Clinical follow-up of participants was not performed as part of these studies, however Oo. et al. undertook a follow-up study of participants from their randomised controlled serological study and found no statistically significant difference in the new case detection rate (NCDR) between the treated, non-treated, and seronegative groups after 6 years [32,33]. The authors concluded that this regimen was ineffective at preventing leprosy and postulated that chemoprophylaxis may have prolonged the incubation period given new cases were not detected for 5 years in the treatment group, compared to 4 years in the seronegative and non-treatment groups. The numbers of new cases were small overall, and the authors did not comment on the risk of re-exposure in those developing leprosy several years post-chemoprophylaxis [45].

Astari et al. investigated the effect of chemoprophylaxis using rifampicin (300 mg/day) and clarithromycin (250 mg/day), given daily for 10 days, followed by a single dose of both medications every 2 weeks for 3 months, on PGL-1 antibody titres in elementary school children in Indonesia. This regimen was associated with a significant decline in PGL-1 antibodies year-on-year over 5 years in school children with PGL-1 IgM antibody seropositivity on screening in a high endemicity area. The regimen was well tolerated and there was no progression to clinical leprosy in any of the children at 5-year follow-up [34]. There was no control group in this study, and children without PGL-1 antibodies on screening were not followed up. This PEP combination has demonstrated superior clinical efficacy compared with any single antibiotic in a nude mouse model of subclinical leprosy [46]. No other large-scale studies using this combination regimen have been performed. However, Hinders et al. have recently published the PEP++ study protocol, a multicentre cluster randomised controlled study designed to explore the efficacy of this enhanced regimen compared to the currently recommended SDR-PEP [35]. Participants will receive three doses of rifampicin (150–600 mg) and clarithromycin (150–500 mg) at 4 weekly intervals and be followed for 2 years.

BE-PEOPLE, a follow-on from the PEOPLE study, is another cluster randomised controlled trial that began in 2023 and will investigate the effectiveness of two doses of bedaquiline (800 mg) and rifampicin (600 mg) given 4 weeks apart as post-exposure prophylaxis compared to SDR-PEP [36]. Bedaquiline has a long half-life, is active against replicating and non-replicating bacteria, and has proven highly effective in the treatment of drug-resistant M. tuberculosis infections.

#### 3.2.3. Safety

Influenza-like syndrome and hepatotoxicity are the main adverse events reported with the use of rifamycin medications. Single-dose rifampicin chemoprophylaxis is generally well tolerated, with minimal or no adverse effects reported in participants in larger controlled studies. In the LPEP study, which had 151,928 participants overall, only three adverse events were reported, and none were considered serious [18]. No adverse events were reported in the PEOPLE study, although active follow-up was not undertaken [26]. Wang et al. did not report any adverse events with use of rifapentine or rifampicin in their study [25]. There is less experience with combination regimens, which might be expected to have an increased risk of medication-related side effects. However, Daulako and Tin reported very low rates of adverse events with ROM prophylaxis in two uncontrolled studies (0.16% and 0.02%, respectively) [30,31].

#### 3.2.4. Promotion of Antimicrobial Resistance

Administration of rifamycin-based chemoprophylaxis could theoretically promote antimicrobial resistance in both *M. tuberculosis* and *M. leprae*. Prior to the implementation of the LPEP study, an expert meeting was held with the aim of producing a consensus statement on the risk of SDR promoting rifampicin resistance. The participants concluded that when combined with screening for TB symptoms, a single dose of medication would have insufficient power to cause the selection of resistant mutants based on known mechanisms of drug resistance in TB. However, molecular monitoring for resistance mutations in both *M. tuberculosis* and *M. leprae* was recommended as part of chemoprophylaxis programmes [47]. A study of drug resistance in *M. Leprae* in Comoros using novel targeted next-generation sequencing did not identify any resistance mutations in patients with leprosy diagnosed between 2017 and 2020. This included patients who had previously been treated with MDT (n = 9) and patients who resided in villages where PEP had been administered as part of the PEOPLE study in 2015 and 2019 (n = 45), two of whom had received PEP. The authors interpreted the results as providing some reassurance that PEP was not selecting resistant strains but acknowledged a larger sample size would be preferable to confirm this finding [48].

#### 3.2.5. Feasibility and Acceptability of Rifamycin-Based Chemoprophylaxis

Ethical concerns have been raised previously with regard to patient confidentiality, the generation of stigma, and the general acceptability of leprosy contact tracing and chemoprophylaxis [19,20]. Qualitative studies, using semi-structured interviews and focus groups, have found that most respondents perceived such interventions positively [49,50,51,52]. Disclosure to household contacts was not a barrier to PEP administration in these studies; however, participants were often unwilling to disclose disease status outside of the immediate household due to concerns regarding stigma. Chemoprophylaxis was generally well-received by study participants, as reflected by low participant refusal rates (1% or less in the largest studies) [7,53]. Misinformation about leprosy was identified as a barrier to the implementation of PEP programmes in India, Nepal, and Indonesia [50].

The feasibility of integrating chemoprophylaxis for contacts into leprosy programmes has been evaluated in different geographic locations using various approaches. The leprosy post-exposure prophylaxis (LPEP) study is the largest feasibility study to date [53]. The study was conducted in seven countries (Brazil, India, Indonesia, Myanmar, Nepal, Sri Lanka, and Tanzania) and showed that SDR-PEP was safe, generally well accepted, and feasible to integrate into national leprosy programmes with little additional effort once contact tracing was established. Retroactive case-finding and PEP administration have also been shown to be feasible and acceptable in low- and high-endemic settings [54,55,56]. In isolated communities with high incidence rates, blanket approaches have been utilised, and this approach was previously explored in Indonesia and is currently being evaluated in Kiribati [28,38].

Re-invigoration of leprosy programmes has been an important benefit of contact tracing and SDR-PEP interventions. Such programmes have positive effects on morale and motivation and can have an important role in enhancing knowledge and capacity building [18,57,58]. Incorporating leprosy screening into broader community skin health activities can help reduce barriers to patients seeking clinical review and have a positive impact on leprosy control efforts [55,59].

#### 3.2.6. Modelling

Mathematical modelling based on data from the COLEP study (SIMCOLEP model) has predicted the efficacy of chemoprophylaxis interventions in various settings. Fischer et al. used a model to investigate seven leprosy intervention scenarios compared with a baseline scenario of passive case detection, MDT, contact tracing, and BCG vaccination of infants. They demonstrated that leprosy incidence would be substantially reduced with a combination of good BCG coverage and early diagnosis, treatment, and chemoprophylaxis of household contacts [60]. Since then, their model has been applied to other leprosy endemic settings globally. In Brazil, SIMCOLEP modelling predicted leprosy elimination could be achieved 2 years earlier with the addition of chemoprophylaxis to current leprosy control activities [61]. Similarly, modelling predicted that intensive population-based chemoprophylaxis together with household chemoprophylaxis would achieve the most rapid and sustained decline in leprosy incidence in Kiribati [62].

Taal et al. applied the SIMCOLEP model to seven endemic leprosy settings and demonstrated the quantity of PEP required to achieve a 90% reduction in the NCDR globally within 22 years. Almost 33 million doses of PEP would be necessary in India, Brazil, and Indonesia alone. A 50% reduction in the global new case detection rate would be achieved within 5 years with intensive contact screening and administration of PEP, assuming 50% efficacy of PEP among household contacts and 70% among other contacts [63].

When applied to the study areas of the Leprosy Post-Exposure Prophylaxis (LPEP) feasibility study, modelling showed that the benefit of the intervention gradually increased over time and could accelerate the timeline of achieving elimination targets by several years [64]. Spatiotemporal mapping of the impact of the LPEP programme activities in Brazil demonstrated that active surveillance of contacts can alter disease patterns and contribute to a reduction in the risk of disease in high-risk areas [65].

#### 3.2.7. Implementation and Cost Effectiveness of Chemoprophylaxis Programmes

Various chemoprophylaxis implementation approaches have been reported in the published literature, each having potential advantages and disadvantages. Guidance outlining the minimal essential dataset required, as well as decision support tools for implementation, have also been published [66,67]. The most commonly used approach is screening close contacts of index cases, which has been employed in high- and low-endemic settings [17,24,68]. The definition of who is considered a close contact needs to be determined based on the socioeconomic and cultural environment in which the intervention is taking place, and some flexibility can be employed to ensure close contacts not meeting a specific definition are not missed. As discussed above, this approach is acceptable with low rates of refusal with regards to disclosure of disease status or taking SDR-PEP [25,49,50,53,55]. This method has been shown to be cost-effective, at least in high-burden countries [69,70]. Economic analysis should be completed for individual countries based on the local costs of delivering care and barriers to implementation, such as geography, population density, and operational costs.

The contact self-screening approach has the potential benefit of increasing the number of contacts screened without requiring additional health-worker resources, while simultaneously increasing community awareness of leprosy signs and symptoms. This approach has not been widely utilised, with some concerns that it would not in fact reduce clinical workloads and might result in missed cases [67]. Stigma could also be a barrier to self-reporting and contribute to under-reporting. Further implementation research is required to assess the efficacy and cost-effectiveness of this approach.

The feasibility of retrospectively tracing contacts of index cases was demonstrated in Cambodia, with drives performed by mobile teams of healthcare workers [56]. Retrospective active case finding (RACF) was found to complement the passive case finding employed in the country at that time but required a well-maintained leprosy database to operate effectively. This approach was subsequently combined with SDR-PEP as part of the LPEP study in Cambodia and was found to be feasible and acceptable [54]. Similar results were noted using this approach in Kiribati, a high endemicity setting, where RACF was implemented at the same time as household contact tracing was introduced in 2018 [55]. In both settings, RACF provided useful gains in terms of new case detection (0.5% of those screened) and could be utilised to enhance leprosy control efforts, particularly if implementing contact tracing and PEP simultaneously. The impact in terms of the number of contacts traced decreased as index case diagnosis and RACF increased. An economic analysis of this approach has not been conducted to date.

In high-endemic settings that are densely populated or geographically isolated, defining close contacts can be difficult. Additionally, including neighbour or social contacts can have implications for patient confidentiality and risk leprosy-associated stigma. In the COLEP study, SDR-PEP was found to be more efficacious in extended contacts, and the PEOPLE study demonstrated that the risk of incident leprosy was two to four times higher in those living within 75 metres of an index case compared to more geographically distant contacts [7,26]. In such situations, a blanket or mass drug administration (MDA) approach may be preferable, as demonstrated by Bakker et al. in Indonesia. This can be population-wide or focus on incidence or population hotspots (focused MDA) [17]. Given the demonstrated feasibility and efficacy of this approach, a population-wide screening and chemoprophylaxis programme for leprosy, combined with tuberculosis screening and treatment, is being implemented in Kiribati, a nation with a high leprosy incidence and a geographically isolated population. This study will include a cost-efficiency analysis [28].

Community skin camps, where patients can self-refer and multiple skin diseases are screened for simultaneously, have been used as part of leprosy control programmes. Integrating leprosy screening with community skin camps has the potential to reduce barriers to seeking clinical review, particularly in communities where stigma is an issue. It does not require disclosure of the index patient’s disease status and simultaneously raises community awareness, particularly if combined with community education and pre-engagement activities. This method has been used previously, but its acceptability or efficacy has not been formally evaluated [71]. One concern is that population coverage may not be sufficient to interrupt disease transmission, particularly in highly endemic settings where there is sustained transmission. The PEP4LEP study is a cluster randomised trial that will compare the efficacy and feasibility of community-based skin camps versus health centre-based screening and SDR-PEP administration for contacts of leprosy in Sub-Saharan Africa [27].

## 4. Discussion

Research reported to date suggests that leprosy post-exposure prophylaxis is effective for reducing the risk of leprosy in contacts of index cases. It appears to be safe, feasible, and acceptable, and in the short term, is the most promising intervention available to interrupt disease transmission when combined with active case detection and MDT treatment. Implementation needs to be tailored to individual settings to maximise effectiveness. Optimisation of chemoprophylaxis regimens is required, particularly for endemic settings and household contacts, and these are areas of ongoing active research. Potential harms relating to chemoprophylaxis appear to be low based on current evidence. However, the risks of stigma, adverse effects, and drug resistance need to be borne in mind in future research as chemoprophylaxis is integrated into national leprosy programmes on a wider scale following WHO recommendations.

The reported efficacy of SDR-PEP is generally considered to be around 50–60% overall. Some argue that this represents insufficient benefit to warrant largescale use as a means of interrupting transmission and does not provide enough individual protection for household contacts, who are at greatest risk [19,20,72]. However, in some circumstances, the efficacy can approach 80%, depending on factors such as the prophylaxis regimen, implementation approach, and history of BCG vaccination [17,25,40]. Despite not showing an overall benefit of double-dose rifampicin over SDR-PEP, the PEOPLE study confirmed a significant protective effect at an individual level (OR 0.55) and an improved protective effect in HHC (OR 0.35) compared to the COLEP study, while noting a significant ongoing risk of disease regardless of regimen used in this population [7,26]. This could suggest an improved efficacy with higher dosing in this high-risk group, but further evaluation is required to confirm this. The recent finding of an enhanced and longer-lasting protective effect in HHC using rifapentine is noteworthy, and strong consideration should be given to its inclusion in future chemoprophylaxis regimens and clinical trials [25,73]. To date, research into other enhanced PEP regimens has been mainly limited to uncontrolled trials and serological studies [29,30,31,32,33,34]. The results of the PEP++ and BE-PEOPLE randomised cluster-controlled studies in the coming years are eagerly awaited [35,36]. Any enhanced efficacy, if demonstrated, will need to be carefully balanced against the potential for additional medication-related adverse effects, increased delivery costs, and risk of resistance developing to other drug classes. Extending chemoprophylaxis interventions to social and neighbour contacts and considering blanket approaches (either MDA or focused MDA) in endemic settings can further improve the effectiveness of this approach and is supported by trial results and modelling studies [17,26,38,62].

The risks and benefits of any intervention need to be weighed at the individual and population level. There is good evidence that chemoprophylaxis is effective, but whether the magnitude of effect warrants exposing significant proportions of communities or populations to chemoprophylaxis medications has been questioned. Other concerns regarding ethical challenges and the promotion of drug resistance have been partially addressed in studies following COLEP. At an individual level, most who receive prophylaxis will not go on to develop clinical leprosy. Ethically, it is important to acknowledge this and the fact that the protective effect of chemoprophylaxis is not absolute and likely to be time-limited when consenting individuals. Currently, there is no reliable diagnostic test to determine who is at risk of developing clinical disease. Target populations are based on the estimated likelihood of exposure [74]. The likelihood of individual benefit of chemoprophylaxis will be affected by the closeness of contact, classification of the index case (PB versus MB), endemic setting, previous BCG vaccination, and characteristics of the index case [17,26,40,74]. Individual and societal benefits complement each other but cannot be easily separated. Based on the low refusal rates reported, the minor individual risks associated with rifamycin-based PEP appear to be palatable to study populations [49,50,51].

Disclosure of disease status to HHCs does not seem to be a barrier to PEP implementation, but gaining consent for this as part of interventions is essential. Alternative measures should be put in place to reach contacts of index cases when initial follow-up is unsuccessful. Ideally, this should include reporting new cases of migrants to their country of origin and contact tracing beyond local and international borders. Extending disclosure beyond the household, while less socially acceptable, can be overcome by taking a community-based or blanket approach depending on the setting [49]. The risk of promoting disease-related stigma appears to be manageable with carefully considered interventions, particularly if complemented by community education and awareness campaigns.

At a broader level, the promotion of drug resistance in both leprosy and TB is a valid concern. The overall risk is considered extremely low and preliminary research has not shown evidence of SDR-PEP promoting the development of resistance [47,48]. However, larger sample sizes and more rigorous testing of patients with leprosy who have had previous exposure to PEP are required to confirm this finding. Resistance to rifampicin, dapsone, ofloxacin, and minocycline has been reported worldwide, highlighting the importance of resistance screening as part of leprosy control programmes [75,76,77]. This is recommended by the WHO and is increasingly becoming a standard component of chemoprophylaxis research studies [5,26,28,36,48]. Combined with adequate TB screening measures, this is essential to minimise risks associated with large-scale PEP interventions and to confirm expert consensus opinion that the risk of promoting resistance is low.

Overall, current evidence favours a net benefit of rifamycin-based PEP at both the individual and population levels through reduced clinical disease and interruption of transmission, with a low risk of harm. Research continues into the optimisation of regimens and implementation strategies, with increasing numbers of countries adopting or considering PEP as part of national leprosy control strategies, in line with WHO recommendations.

Integration of chemoprophylaxis programmes into the strategic plans of leprosy programmes is key to the effectiveness and sustainability of this approach. The LPEP study has demonstrated feasibility in diverse geographical settings and incurs little additional cost once contact tracing has been established [53]. Economic analyses of all available implementation approaches have not been conducted to date, and each has advantages and disadvantages which must be tailored to the individual epidemiological, sociocultural, and economic setting in which it will be implemented. Blanket approaches may be preferable in high endemicity settings but come at increased cost and may not always be financially viable [17,26]. While the COLEP study suggested reduced efficacy of PEP in HHCs, the PEOPLE study demonstrated a better protective effect in this group using a higher dose of rifampicin. The number needed to treat was 10 times lower compared to the overall study population, which has important implications for intervention-related costs. Rifapentine has already been demonstrated to have superior efficacy to SDR in reducing clinical disease in HHCs out to 4 years, and combination regimens are being assessed in the PEP++ and BE-PEOPLE studies. Targeting HHC with an enhanced regimen may represent a better return on investment in areas of high incidence where a blanket approach is not possible for cultural, operational, or financial reasons. It is possible that alternative regimens for different levels of contact could be considered within the same programme, with an enhanced regimen for HHC and continuation of SDR for extended contacts as current standard practice. Geospatial analysis and modelling may be useful tools to inform the appropriate breadth of contact tracing and chemoprophylaxis in individual settings, with the potential to improve programme efficiency and cost-effectiveness [8]. The development of tools to aid early diagnosis and identification of contacts at the highest risk of acquisition of leprosy is an area of ongoing research that may facilitate more targeted chemoprophylaxis strategies in the future. Currently available diagnostics do not support such approaches.

An important, and possibly under-recognised consequence of chemoprophylaxis programmes is the reinvigoration of leprosy control efforts, and this impact should not be underestimated. Leprosy disproportionally affects the most disadvantaged populations. Leprosy programmes have often been overlooked and underfunded, with a lack of commitment to long-term resourcing. This has led to programmes defaulting to a passive case-finding model of care that contributes to continued disease transmission in endemic countries. Chemoprophylaxis programmes have been positively received by healthcare staff, people affected by leprosy, and communities [49,51,53]. Improved morale associated with the availability of an acceptable intervention was noted across all countries that participated in the LPEP study regardless of the baseline leprosy control efforts in place prior to the study. Improved healthcare staff training and knowledge, as well as improved community awareness of leprosy, contributes to reduced disease-related stigma [53,59,78].

## 5. Conclusions

Leprosy chemoprophylaxis has been shown to be a safe and effective component of leprosy control programmes with benefits that extend beyond the individual through the invigoration of leprosy control efforts and increased awareness. Additional research is needed to optimise PEP regimens while ensuring adequate monitoring for potential intervention-related harm, especially as more active agents such as bedaquiline, rifapentine, and combination regimens are assessed. Evidence gathered to date has been incorporated into the design of newer randomised trials, which will provide further information on the best means of implementing chemoprophylaxis to maximise benefit and efficiency. The addition of PEP to existing active case finding and MDT efforts currently offers the greatest hope of interrupting *M. leprae* transmission and thus propelling endemic countries towards the goal of zero leprosy.

## Supplementary Materials

The following supporting information can be downloaded at: https://www.mdpi.com/article/10.3390/tropicalmed10040084/s1, Figure S1: Risk of bias assessment for four main controlled studies on efficacy of chemoprophylaxis.

## Abbreviations

SDR: Single dose rifampicin; SDDR: Single double dose rifampicin; PEP: Post-exposure prophylaxis; HHC: Household contacts; MDT: Multidrug therapy; MDA: Mass drug administration; ROM: Rifampicin, Ofloxacin, Minocycline; BCG: Bacillus Calmette-Guérin; *M. leprae*: *Mycobacterium leprae*; *M. tuberculosis*: *Mycobacterium tuberculosis*; PGL-1: anti-phenol glycolipid-1; IRR: Incidence rate ratio; HR: Hazard ratio; NNT: Number needed to treat; OR: Odds ratio; CI: Confidence Interval.
