# Supplementary material for: A Review of the Efficacy, Safety, and Feasibility of Rifamycin-Based Post-Exposure Chemoprophylaxis for Leprosy
Source: Trop Med Infect Dis. 2025 Mar 21;10(4):84. doi: 10.3390/tropicalmed10040084 (PMC12030903; doi:10.3390/tropicalmed10040084)
Supplement: Supplementary file 1 [file tropicalmed-10-00084-s001.zip › tropicalmed-3522863-supplementary.pdf]

Supplementary Material

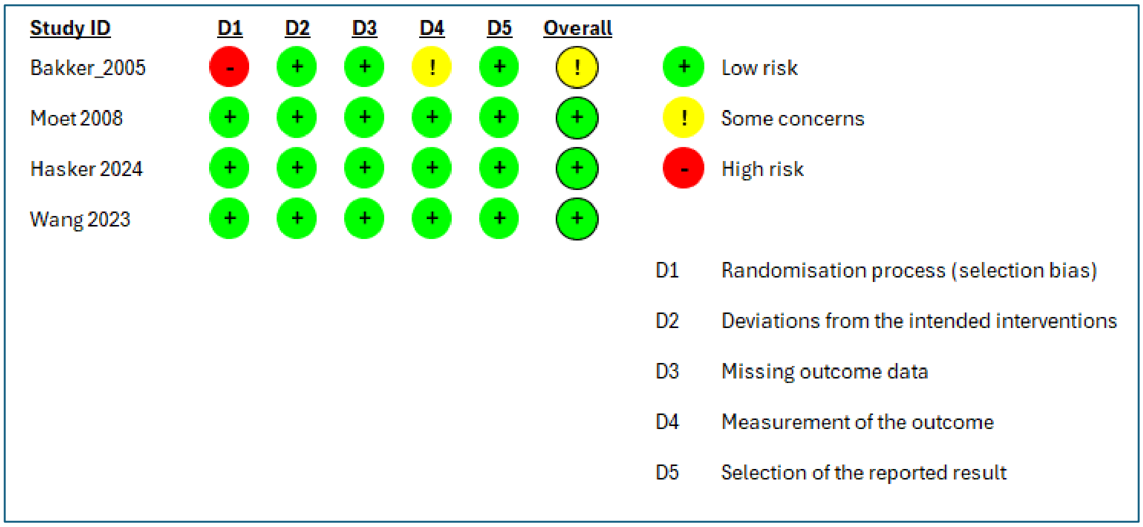

Supplementary Figure S1. Risk of bias assessment for four main controlled studies on efficacy of chemoprophylaxis
